# Factors Associated with the Retinal Nerve Fiber Layer Loss after Acute Primary Angle Closure: A Prospective EDI-OCT Study

**DOI:** 10.1371/journal.pone.0168678

**Published:** 2017-01-31

**Authors:** Eun Ji Lee, Tae-Woo Kim, Kyoung Min Lee, Seung Hyen Lee, Hyunjoong Kim

**Affiliations:** 1 Department of Ophthalmology, Seoul National University College of Medicine, Seoul National University Bundang Hospital, Seongnam, Korea; 2 Department of Applied Statistics, Yonsei University, Seoul, Korea; Charite Universitatsmedizin Berlin, GERMANY

## Abstract

**Purpose:**

To determine the factors associated with retinal nerve fiber layer (RNFL) loss in eyes with acute primary angle-closure (APAC), particularly focusing on the influence of the change in the anterior lamina cribrosa surface depth (LCD).

**Methods:**

After the initial presentation, 30 eyes with unilateral APAC were followed up at the following specific time points over a 12-month period: 1 week, 1~2 months, 2~3 months, 5~6 months, and 11~12 months. These follow-ups involved intraocular pressure measurements, enhanced depth-imaging spectral-domain optical coherence tomography (SD-OCT) scanning of the optic disc, and measurements of the circumpapillary RNFL thickness. The prelaminar tissue thickness (PLT) and LCD were determined in the SD-OCT images obtained at each follow-up visit.

**Results:**

Repeated measures analysis of variance revealed a significant pattern of decrease in the global RNFL thickness, PLT, and LCD (all *p*<0.001). The global RNFL thickness decreased continuously throughout the follow-up period, while the PLT decreased until 5~6 months and did not change thereafter. The LCD reduced until 2~3 months and then also remained steady. Multivariable regression analysis revealed that symptoms with a longer duration before receiving laser peripheral iridotomy (LI) (*p* = 0.049) and a larger LCD reduction (*p* = 0.034) were significant factors associated with the conversion to an abnormal RNFL thickness defined using OCT normative data.

**Conclusion:**

Early short-term decreases in the PLT and LCD and overall long-term decrease in the peripapillary RNFL were observed during a 12-month follow-up after an APAC episode. A longer duration of symptoms before receiving LI treatment and larger LCD reduction during follow-up were associated with the progressive RNFL loss. The LCD reduction may indicate a prior presence of significant pressure-induced stress that had been imposed on the optic nerve head at the time of APAC episode. Glaucomatous progression should be suspected in eyes showing LCD reduction after the APAC remission.

## Introduction

Acute primary angle closure (APAC) is characterized by an acute, symptomatic intraocular pressure (IOP) increase followed by rapid recovery from the symptoms after applying timely treatment. Although APAC generally does not result in significant visual morbidity if it is treated promptly, delayed optic nerve damage still can occur even after the remission of acute episodes.[1–5] However, the mechanism underlying the development of permanent optic nerve damage even after the acute attack has resolved and the initiating or influencing factors are yet to be established.[2, 6, 7]

Changes in the optic nerve head (ONH) in APAC have been described as an initial swelling and hyperemia that resolves after applying timely treatment, or is followed by pallor of the optic disc with diffuse thinning of the axons when the acute episode leads to permanent sequelae in the ONH.[8–10] However, changes in the deep ONH tissues (i.e., prelaminar tissue or the lamina cribrosa [LC]) or peripapillary tissues (i.e., peripapillary choroid) after APAC are not well described. The interplay between the deep ONH tissues and axons has been considered a key to understanding the mechanism of glaucomatous optic neuropathy, which has prompted explorations of the deep ONH tissues including the LC.[11, 12] Numerous experimental and clinical studies have suggested that structural changes in the LC and peripapillary tissues have significant implications for glaucoma pathogenesis.[12–23] However, most of these studies have focused on open-angle glaucoma (OAG), with few investigating eyes with APAC[24] or suspected APAC diagnosed based on the darkroom-prone provocative test.[25]

The aim of the study was to elucidate longitudinal changes in the deep ONH and peripapillary tissues after an APAC episode, and to correlate them with the development of RNFL loss after APAC remission.

## Methods

### Study Design

This investigation was based on the data of APAC patients obtained in an ongoing prospective study of patients with glaucoma and healthy subjects being conducted at the Seoul National University Bundang Hospital Glaucoma Clinic, Seoul, South Korea (the Angle Closure Glaucoma Prognosis Study). All of the participants provided written informed consent. This study was approved by the Seoul National University Bundang Hospital Institutional Review Board and followed the tenets of the Declaration of Helsinki.

### Study Participants

To be included in this study, patients had to have been diagnosed with APAC and had been followed up for at least 12 months after the remission of the APAC episode. The APAC diagnosis was based on the following criteria:[2, 8] (1) presence of at least two of the following symptoms: ocular pain, headache, blurred vision, and nausea and/or vomiting; (2) presenting IOP of >21 mmHg (as measured by Goldmann applanation tonometry); and (3) Presence of at least three of the following signs: conjunctival injection, corneal epithelial edema, mid-dilated unreactive pupil, and shallow anterior chamber. Participants received medical treatment followed by laser peripheral iridotomy (LI) within 12 hours of the documented APAC. If both eyes of a patient were eligible for inclusion, one of these eyes was randomly selected.

The following exclusion criteria were applied: (1) secondary angle closure, such as lens-induced glaucoma, neovascular glaucoma, or uveitic glaucoma; (2) pre-existing diagnosis of any forms of glaucoma, (3) presence of coexisting retinal or neurologic disease; (4) history of previous intraocular surgery, or intraocular surgery during the follow-up period; (5) poor-quality spectral-domain optical coherence tomography (SD-OCT) scans of the optic disc (i.e., quality score <15) in more than 5 sections that did not allow precise measurement of the ONH parameters (when the quality score does not reach 15, the image acquisition process performed by the Spectralis OCT system automatically stops, and images of the respective sections are not obtained); and (6) best-corrected visual acuity of <20/50 after remission.

Forty-two APAC patients who met the eligibility criteria were initially recruited. Of these, 12 were excluded due to the poor scan quality preventing precise measurements of the ONH parameters (see *Measurements of the ONH Parameters*). Table 1 presents the clinical characteristics of the 30 participants, who comprised 22 women and 8 men aged 65.6±7.7 years (mean±standard deviation; range, 51 to 82 years). The participants had a best-corrected visual acuity ranging from 20/50 to 20/15 after APAC remission, a refractive error (spherical equivalent) of 1.0±1.7 diopters (range, –3.25 to +3.63 diopters), and an axial length of 22.4±0.8 mm (range, 21.1 to 23.9 mm; Table 1).

**Table 1:** Patients’ clinical characteristics.

Age, *years*	65.6 ± 7.7 (51 to 82)
Gender (male/female), *n*	8 / 22
Duration of symptoms until LI treatment, *hours*	16.3 ± 19.6 (1 to 90)
Presenting IOP, *mmHg*	48.1 ± 10.5 (29 to 70)
Post-LI IOP, *mmHg*	13.6 ± 5.6 (7 to 33)
Presenting best-corrected visual acuity, *LogMAR*	1.0 ± 0.7 (0.1 to 2.6)
Best-corrected visual acuity after remission, *LogMAR*	0.2 ± 0.1 (-0.1 to 0.4)
Refractive error, *diopters*	1.0 ± 1.7 (-3.25 to 3.63)
Axial length, *mm*	22.4 ± 0.8 (21.1 to 23.9)
Central corneal thickness, *μm*	577.5 ± 30.0 (525 to 657)

Values are given as mean ± standard deviation (range, min to max) unless otherwise specified.

LI = laser iridotomy; IOP = intraocular pressure; LogMAR = log of the minimum angle of resolution

### History-Taking and Ocular Examinations

History-taking and ocular examinations were applied at the time of first presentation and/or 1 week after the remission of the APAC episode in cases where an ocular examination could not be performed due to the presence of corneal edema during the acute episode. The patient history included demographic characteristics, duration of symptoms (pain, headache, nausea/vomiting, and blurred vision), administration of oral medications, and other systemic diseases. Ocular examinations included visual acuity assessment, Goldmann applanation tonometry, refraction tests, slit-lamp biomicroscopy, gonioscopy, and undilated stereoscopic examination of the optic disc. The patients also underwent color fundus photography (EOS D60 digital camera, Canon, Utsunomiyashi, Tochigiken, Japan), SD-OCT circumpapillary retinal nerve fiber layer (RNFL) scanning, SD-OCT optic disc scanning using the enhanced depth-imaging (EDI) technique (Spectralis, Heidelberg Engineering, Heidelberg, Germany), and measurements of corneal curvature (KR-1800, Topcon, Tokyo, Japan), central corneal thickness (Orbscan II, Bausch & Lomb Surgical, Rochester, NY, USA), and axial length (IOL Master v. 5, Carl Zeiss Meditec, Dublin, CA).

The participants were followed up at 1 week (FU1), 1~2 months (FU2), 2~3 months (FU3), 5~6 months (FU4), and 11~12 months (FU5) after the initial visit. At each follow-up the participants underwent visual acuity assessment, Goldmann applanation tonometry, slit-lamp biomicroscopy including evaluation of the peripheral anterior chamber depth using the van Herick technique,[26] undilated stereoscopic examination of the optic disc, and SD-OCT examination.

The IOP was recorded at the time of the APAC onset, within 30 minutes after LI, and then at every follow-up visit until FU5. The mean and standard deviation values of the follow-up IOP measurements were defined as the mean follow-up IOP and the IOP fluctuation, respectively. IOP re-elevation during the subsequent follow-up was defined when the IOP increased to >21mmHg.

### SD-OCT Scanning of the Optic Disc and Circumpapillary RNFL Thickness Assessment

The ONH was imaged using the Spectralis SD-OCT system with the EDI technique. The details of the protocol for scanning of the optic nerve using EDI SD-OCT to evaluate the LC are described elsewhere.[27, 28]

At the time of ONH scanning, the circumpapillary RNFL thickness was also measured using a circular scanning protocol, the details of which have been published elsewhere.[29, 30] The diameter of the scan circle spanned 12 degrees, with the diameter in millimeters depending on the axial length. The software provided with the Spectralis SD-OCT system provided a global average, and the mean thicknesses determined in each of six sectors obtained by dividing the scan circle into nasal-superior (90–135°), nasal (135–225°), nasal-inferior (225–270°), temporal-inferior (270–315°), temporal (315–45°), and temporal-superior (45–90°) sectors. The global and six sectoral RNFL thicknesses are presented using circular diagrams. In obtaining the circumpapillary RNFL scanning, repeat scan protocol was used, so that the scan circle can be centered on the same ONH location throughout the follow-up period. Progressive RNFL loss or conversion to an abnormal RNFL thickness was defined when any of the six sectors in the circular diagram showed conversion of the RNFL thickness from falling within normative database to beyond the lower 99% confidence limit (red color), and where the RNFL loss was evident in the corresponding area of the circumpapillary B-scan image. Images with failure in the RNFL segmentation were excluded from the analysis.

### Measurements of the ONH Parameters

This study measured the prelaminar tissue thickness (PLT), the anterior LC surface depth relative to the Bruch’s membrane (BM) opening (BMO) level (LCD), the juxtapapillary choroidal thickness (JCT), and the BMO diameter on the EDI SD-OCT scans that had been obtained at each visit. Measurements were also performed for contralateral eyes when SD-OCT results were available for both eyes.

**Fig 1:**
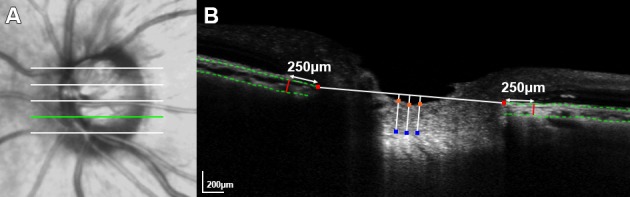
Measurement of the optic nerve head and peripapillary parameters. **(A)** Infrared fundus image of an eye with acute primary angle closure, indicating the locations where the measurements were made. **(B)** Enhanced depth-imaging spectral-domain optical coherence tomography image obtained at the location indicated by the *light-green line* in (A). The anterior lamina cribrosa surface depth (LCD) was determined by measuring the distance from the Bruch’s membrane (BM) opening reference plane (*horizontal white line* connecting the two BM termination points as indicated by *red glyphs*) to the level of the anterior lamina cribrosa (LC) surface at the three most depressed points (*blue glyphs*). The prelaminar tissue thickness was determined as the distance between the optic cup surface (*orange glyphs*) and the anterior LC border (*blue glyphs*), as measured at the three points that were used to measure the LCD. The juxtapapillary choroidal thickness was defined as the perpendicular distance between the BM and the choroidoscleral interface (*light-green dashed lines*), and measured at 250 μm both from the nasal and temporal BM termination points (*red lines*).

The PLT, LCD, and JCT were measured in five horizontal B-scans (superior, superior midperiphery, center, inferior midperiphery, and inferior regions) that divided the optic disc into six equal parts vertically (Fig 1).

The LCD was determined by measuring the distance from the BMO plane to the level of the anterior LC surface.[31–33] A reference line connecting the two termination points of BM was drawn on each B-scan image. The distance from the reference line to the level of the anterior border of the LC was measured at three points: the maximally depressed point and two points that were 100 and 200 μm from the maximally depressed point in a temporal direction. The LC was evident as a hyperreflective plate-like structure in the EDI SD-OCT images, and its anterior border was readily discernible. The PLT was measured at the same three points in the five horizontal B-scans as the LCD,[33] and was determined as the distance between the optic cup surface and the anterior LC border in the direction perpendicular to the optic cup surface. The measurements made at the three points were used to calculate the mean LCD and PLT of the B-scan, and those obtained from the five B-scan images were used to calculate the mean LCD and PLT of the eye.

Because the LCD measured from the BMO level is influenced by the JCT,[34, 35] we speculated that the size of the LCD change could also be biased by changes in the JCT during the study period. Hence, the JCT was also measured at each follow-up in the same five images that were used to measure the LCD. The JCT was measured on both the nasal and temporal sides at 250 μm from the nasal and temporal BM termination points, respectively, and defined as the perpendicular distance between the BM and the choroidoscleral interface (Fig 1). The mean of the nasal and temporal JCTs was defined as the JCT of the B-scan, and the values obtained from the five B-scan images were used to calculate the mean JCT of the eye.

Measurements of the PLT, LCD, and JCT at each time point were performed using the manual caliper tool of the Spectralis viewer (Heidelberg Eye Explorer software v. 1.7.0.0, Heidelberg Engineering), in as close to the same plane as possible. The low reflective shadow within the LC as well as the choroidal shadow was examined to confirm the correspondence of the B-scan image series between images.[31, 33]

The BMO diameter was defined as the mean of the longest and shortest BMO diameters. The longest and shortest BMO diameters were the longest and shortest distances between the two termination points of the BM, which were measured on the radial B-scan images constructed from the 3 dimensional data set of EDI SD-OCT scan. Measurements were made using the manual caliper tool of the Amira 5.2.2 software (Visage Imaging, Berlin, Germany).

All measurements were performed by two independent observers (E.J.L. and K.M.L.) who were blinded to the clinical information of the participants, including the IOP and the time point of scanning for the follow-up SD-OCT images. Measurements were repeated twice by the two observers, and the mean of the four values was used for the main analysis.

### Data Analysis

The interobserver reproducibility for the measurements of ONH parameters and JCT was evaluated using the intraclass correlation coefficients.

Repeated-measures multivariable and univariable analysis of variance as well as paired samples *t*-tests were used to assess changes in ONH measurements during the follow-up. Repeated-measures *p* values were calculated using the Greenhouse-Geisser correction. Intereye comparisons were performed using paired-samples *t*-tests. A logistic regression analysis was performed to investigate the factors associated with the conversion to an abnormal OCT RNFL thickness.

Analyses were performed using the Statistical Package for the Social Sciences (version 20.0, SPSS, Chicago, IL, USA). Unless stated otherwise, the data are presented as mean±standard deviation values, and the cutoff for statistical significance was set at *p*<0.05.

## Results

The duration of symptoms associated with the APAC episode recorded dependent on the patient history was 16.3±19.6 hours (range, 1 to 90 hours; median, 14 hours; Table 1). The IOP was significantly reduced from 48.1±10.5 mmHg (range, 29 to 70 mmHg) at the time of the APAC diagnosis, to 13.6±5.6 mmHg (range, 7 to 33 mmHg) at 30 minutes after LI treatment (P<0.001) (Table 1).

Six patients exhibited a re-elevation of IOP to >21mmHg during the follow-up period: this occurred at FU1 (i.e., 1 week) in three patients and at FU2 (i.e., 1~2 months) in the other three. However, IOP normalized in these patients after the cataract surgery. Twenty-five of the 30 eyes received cataract surgery during the follow-up period according to the patients’ preference, once they had been informed about the effects of lens extraction on the anterior chamber angle anatomy and IOP control in eyes with primary angle closure.[36–38] None of the patients were required to be treated with IOP lowering medication.

EDI SD-OCT optic disc scanning could not be performed at the time of the APAC attack due to corneal edema in all except one eye, and the circumpapillary RNFL thickness could only be evaluated in six eyes. Thus, the SD-OCT measurements made 1 week after the APAC attack were considered as the baseline values for the longitudinal evaluation. The interobserver agreements for the PLT, LCD, JCT, and BMO diameter measurements were good, with intraclass correlation coefficients of 0.978, 0.991, 0.931, and 0.967, respectively.

**Fig 2:**
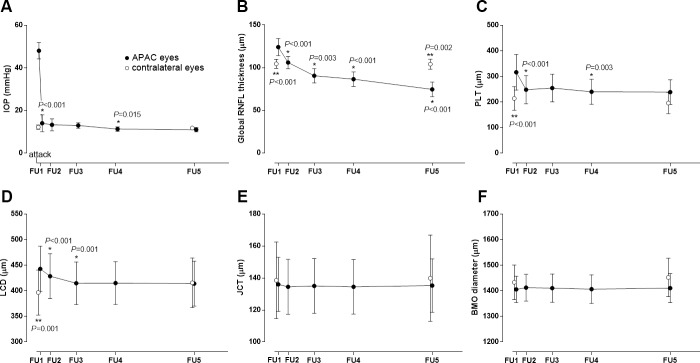
Time courses of changes in the intraocular pressure, and the optic nerve head and peripapillary parameters after an APAC attack in APAC eyes (*closed circles*) and contralateral eyes (*open circles*). The presented data are mean and 95% confidence intervals. *Asterisks* indicate significant changes relative to before follow-up, and *double asterisks* indicate significant intereye differences at each follow-up. Participants were followed up at 1 week (FU1), 1~2 months (FU2), 2~3 months (FU3), 5~6 months (FU4), and 11~12 months (FU5). *APAC = acute primary angle closure; IOP = intraocular pressure; RNFL = retinal nerve fiber layer; PLT = prelaminar tissue thickness; LCD = anterior lamina cribrosa surface depth; JCT = juxtapapillary choroidal thickness; BMO = Bruch’s membrane opening*.

Fig 2 illustrates the time courses of the IOP change during the follow-up, and the changes in the global RNFL thickness and ONH measurements from FU1 to FU5. The global RNFL thickness gradually decreased from FU1 to FU5, with the thinning being more prominent during the early follow-ups (FU2 and FU3) (Fig 2A). In the six patients where the circumpapillary RNFL scanning could be performed at the time of the APAC attack, the global RNFL thickness was significantly increased at FU1 relative to the initial visit (124.2±26.9 *vs*. 118.5±34.5 μm, *p* = 0.031; Wilcoxon signed-rank test). The PLT showed an initial decrease at FU2 and a further decrease at FU4, and then remained steady until FU5 (Fig 2B). The LCD was significantly reduced at FU2 and FU3, and did not change thereafter (Fig 2C). There was no change in the JCT or BMO diameter during the follow-up period (Fig 2E and 2F). Both repeated-measures univariable and multivariable analyses of variance showed significant decreases in the global RNFL thickness, PLT, and LCD from FU1 to FU5 (all *p*<0.001), while neither JCT and nor the BMO diameter showed a significant change (*p* = 0.530 and 0.857, respectively).

Data for contralateral eyes obtained at FU1 and FU5 were used as controls to compare with those of the APAC eyes. In the contralateral eyes, none of the parameters—IOP (*p* = 0.195), global RNFL thickness (*p* = 0.717), PLT (*p* = 0.604), LCD (*p* = 0.581), JCT (*p* = 0.949), or BMO diameter (*p* = 0.514)—differed between FU1 and FU5. The intereye comparisons revealed that the global RNFL thickness was larger in the APAC eyes at FU1 (*p*<0.001) but gradually decreased during the follow-up and became significantly thinner at FU5 (*p* = 0.002, Fig 2B). PLT was also larger in APAC eyes at FU1 (*p*<0.001), while the difference disappeared at FU5 (*p* = 0.144, Fig 2C). LCD was larger in the APAC eyes than in the contralateral eyes at FU1 (*p* = 0.001), but it decreased during the follow-up and became comparable to the LCD of the contralateral eyes (*p* = 0.581, Fig 2D). On the other hand, the IOP (Fig 2A), JCT (Fig 2E), and BMO diameter (Fig 2F) did not show significant intereye differences at FU1 and FU5. All of the data for the APAC and contralateral eyes are provided in the supporting information file (S1 Table).

At FU5, the SD-OCT circumpapillary RNFL thickness measurements revealed a conversion to an abnormal OCT RNFL thickness in 13 out of 30 eyes (43.3%). Logistic regression analysis showed that the conversion to an abnormal OCT RNFL thickness was significantly associated with a longer duration from the symptom onset to receiving LI treatment [odds ratio (OR) = 1.155, *p* = 0.013], re-elevation of IOP after first LI (OR = 12.143, *p* = 0.035), and a larger LCD reduction between the initial visit and FU5 (OR = 1.169, *p* = 0.008) in the univariable analysis (Table 2). In the multivariable analysis, the duration of symptoms (OR = 1.140, *p* = 0.049) and the LCD reduction (OR = 1.164, *p* = 0.034) were significant factors.

**Table 2:** Factors associated with the progressive retinal nerve fiber layer loss after remission of acute primary angle closure (*n* = 30).

	Univariable			Multivariable		
	OR	95% CI	*p*	OR	95% CI	p*
Age, *per 1 year older*	0.972	0.881, 1.072	0.565			
Female gender	1.939E9	0.000, .	0.999			
Duration between the symptom onset and LI, *per 1 hour longer*	**1.155**	**1.031, 1.293**	**0.013**	**1.140**	**1.000, 1.299**	**0.049**
Visual acuity at attack, *per 1LogMAR increase*	1.755	0.633, 4.865	0.280			
Refractive error, *per 1D higher*	1.219	0.779, 1.907	0.385			
Axial length, *per 1 mm longer*	0.670	0.241, 1.864	0.670			
Central corneal thickness, *per 1 μm increase*	1.011	0.986, 1.038	0.383			
IOP at attack, *per 1 mmHg increase*	1.052	0.975, 1.135	0.193			
%IOP reduction after LI, *per 1 mmHg increase*	1.030	0.970, 1.094	0.341			
IOP re-elevation	**12.143**	**1.193, 123.618**	**0.035**	3.668	0.022, 607.595	0.618
Global RNFL thickness at FU1, *per 10 μm increase*	0.876	0.647, 1.184	0.389			
PLT at FU1, *per 10 μm increase*	0.986	0.946, 1.028	0.502			
PLT decrease, *per 1 μm increase*	0.998	0.989, 1.008	0.732			
LCD at FU1, *per 10 μm increase*	1.009	0.948, 1.074	0.785			
LCD decrease, *per 1 μm increase*	**1.169**	**1.041, 1.313**	**0.008**	**1.164**	**1.012, 1.338**	**0.034**
BMO diameter at FU1, *per 100 μm increase*	0.732	0.395, 1.357	0.322			
BMO diameter change, *per 1 μm increase*	1.007	0.993, 1.020	0.348			
JCT at FU1, *per 10 μm increase*	0.863	0.713, 1.046	0.134			
JCT change, *per 1 μm increase*	0.945	0.846, 1.055	0.311			

* Variables with *P* < .10 in the univariable analysis were included in the multivariable analysis.

OR = odds ratio; CI = confidence interval; LI = laser iridotomy; LogMAR = log of the minimum angle of resolution; IOP = intraocular pressure; RNFL = retinal nerve fiber layer; FU1 = 1 week after acute primary angle closure attack; PLT = prelaminar tissue thickness; LCD = anterior lamina cribrosa surface depth; BMO = Bruch’s membrane opening; JCT = juxtapapillary choroidal thickness.

**Fig 3:**
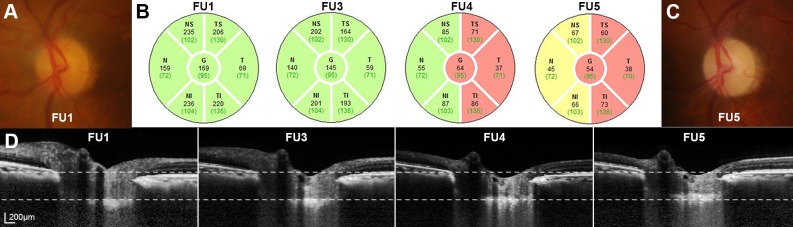
Representative case with acute primary angle closure where the progressive RNFL loss was accompanied by the reversal of the LC. **(A)** Color disc photographs obtained at FU1. **(B)** Serial global and sectorial RNFL thickness measurements made using spectral-domain optical coherence tomography, showing progressive RNFL thinning. **(C)** Color disc photographs obtained at FU5. Note the increased cupping and pallor of the optic nerve head in both eyes. **(D)** Serial B-scan images obtained at the same locations at each follow-up. Superior and inferior *dashed lines* indicate the level of nasal Bruch’s membrane termination points and the level of anterior LC surface at FU1. Note that the anterior LC surface depth decreased gradually during the follow-up. Eyes were followed up at 1 week (FU1), 1~2 months (FU2), 2~3 months (FU3), 5~6 months (FU4), and 11~12 months (FU5). *RNFL = retinal nerve fiber layer; LC = lamina cribrosa*. *Sectors*: *nasal-superior (NS*, *90–135°)*, *nasal (N*, *135–225°)*, *nasal-inferior (NI*, *225–270°)*, *temporal-inferior (TI*, *270–315°)*, *temporal (T*, *315–45°)*, *and temporal-superior (TS*, *45–90°)*. *G*, *global*.

Fig 3 shows the representative case of APAC where the SD-OCT RNFL thickness progressively decreased during the follow-up. The reduction of LCD is notable at FU5 relative to FU1.

## Discussion

The present study has revealed longitudinal changes in the deep ONH tissues and peripapillary RNFL that are present for 1 year after an APAC episode. A short-term reduction of the PLT and LCD during the early follow-up period, and a long-term sustained decrease in the RNFL thickness during the 1-year follow-up were noted after an APAC attack. Eyes with a longer duration from the symptom onset to receiving LI treatment, and a larger LCD reduction during the follow-up were more likely to show progressive RNFL loss. To the authors’ knowledge, this is the first study to show longitudinal changes in the deep ONH tissues after APAC and their influence on the progressive RNFL loss.

The initial increase followed by a subsequent decrease in the peripapillary RNFL thickness after APAC has been well described.[8, 39–41] It has been suggested that these features respectively reflect ONH swelling and its resolution in the course of an APAC episode. An initial increase in the peripapillary RNFL was also observed in the present study, although the data could not be obtained in all participants due to the presence of corneal edema at the time of the APAC attack. In the six eyes where the circumpapillary RNFL scan was obtainable at the time of attack, the global RNFL thickness increased significantly from 118.50±34.53 to 129.19±36.98 μm at FU1 (*P* = 0.028, Wilcoxon signed-rank test), and then exhibited a continuous decrease from FU1 to FU5, with 40% of the participants showing RNFL loss below the normal range.

The PLT decreased notably during the earlier follow-ups, but did not change at later follow-ups. Given that both the RNFL and prelaminar tissue consist mainly of retinal ganglion cell axons, thinning of the RNFL is thought to accompany a decrease in the PLT. However, this was not true in the later follow-up, when RNFL thinning was not associated with any significant PLT change. This finding is in accordance with a previous finding of the absence of optic disc cupping despite thinning of the RNFL after APAC resolution.[10] We attributed the discrepancy between the RNFL and PLT change in the later follow-up to the reactive gliosis accompanied by ischemia or inflammation of the ONH associated with acute IOP change.[42–44] It is relevant that the PLT decreases in the earlier follow-up with resolution of the ONH swelling. However, when the swelling is absorbed, the PLT does not necessarily decrease despite ongoing RNFL thinning, because of the reactive gliosis. This reactive gliosis may compensate for the loss of neural components in the prelaminar tissue that should have occurred along with the axonal degeneration.

In the present study, the LCD reduced significantly during the earlier follow-ups and then did not change during the later follow-ups. This is comparable to the pattern of longitudinal change in the LCD after trabeculectomy in OAG patients.[33] It is intriguing that the LCD reduction was associated with the progressive RNFL loss. We previously speculated that the reduction of the LCD after IOP-lowering treatment observed in OAG could reflect the reduced IOP-related stress on the LC.[31, 33] Indeed, we recently found that a long-term sustained reduction in the LCD is important to slowing the disease progression in OAG eyes.[22] However, this did not seem to be the case for the APAC patients in the present study. We speculate that the LCD reduction reflects significant displacement of the LC during the APAC episode. The displacement may have disrupted the structural and/or functional integrity between the laminar beams and axons. The RNFL loss after APAC remission despite the LCD reduction suggests that axonal damage occurs in a sustained fashion once the integrity is disrupted. In contrast, no (or less) LCD reduction after IOP lowering may be an indicator that the LC had not been (or was less) displaced during the APAC attack. The lack of RNFL damage in these cases supports this speculation.

It is noteworthy that the LCD reduction at final FU was smaller in APAC patients than in the OAG eyes included in our previous study (28.84 *vs*. 110.68 μm),[33] despite the IOP reduction being larger in the present study (37.1 *vs*. 16.7 mmHg). This may be attributable to the initial disc scan being performed 1 week after LI treatment, when some LCD reduction might have already occurred. The EDI SD-OCT images were not obtainable at the time of the APAC attack in most of the patients due to the presence of corneal edema, which is one of the main limitations of this study (see below). It is also possible that because the IOP elevation is highly symptomatic in APAC, and also decreases rapidly, the duration of IOP elevation is shorter in APAC than in chronic glaucoma, and such a short-term IOP elevation may not induce a large LC displacement as is observed in chronic glaucoma. Alternatively, acute IOP lowering may have induced shrinkage of the scleral canal, leading to the relief of the tensile force that had been stretching the LC within the scleral canal, which in turn moved the LC posteriorly and thus lessened the LCD reduction.[13, 45] Although the BMO diameter did not change during the follow-up in the present study, this could have been due to the lack of EDI SD-OCT images obtained at the time of the APAC attack. On the other hand, mechanical properties or geometry of eyeball might differ between APAC and OAG eyes, which might have resulted in differed response of the LC.

It has been suggested that the LCD measured from the BMO level is influenced by the JCT,[34, 35] such that eyes with a thick choroid would have a larger LCD than those with a thin choroid. Thus, LCD data should be interpreted cautiously when the JCT varies. In the present study, no significant change was found in the JCT among the follow-ups, suggesting that the JCT had a negligible influence on the estimation of the LCD change.

While the results have been equivocal and variable, a longer duration of symptoms,[4, 5, 9] greater IOP increase,[3] larger CD ratio at baseline,[5] and worse initial visual field[3] have been suggested as potential risk factors for glaucoma progression after APAC. Together with the LCD reduction, the present study found a significant influence of the duration from the symptom onset to receiving LI treatment. This finding emphasizes the importance of early presentation and rapid normalization of IOP to prevent the development of glaucoma. However, factors that were not evaluated in the study may also have contributed to glaucomatous damage. It has been suggested that abrupt IOP elevation may induce total blockade of the ONH blood flow and axonal transport, causing irreversible damage to the axons.[46–48] Together with the damage associated with the LC change, the direct effect of IOP elevation and secondary axonal degeneration may also play a role in glaucoma development in APAC patients. The development of glaucoma in APAC is likely to be influenced by a complex multiple factors. Further study using more advanced imaging tools may help to reveal these factors and how they are related to glaucoma in APAC.

In contrast to the findings of this study, Jiang et al.[25] did not find any significant changes in the LCD with IOP elevation in patients with suspected APAC. However, these two studies cannot be compared directly due to the inclusion of different participants and differences in the size and duration of the IOP changes. In addition, that previous study found ONH changes for short-term IOP elevation, while the present study observed long-term changes after IOP lowering.

This study was subject to several limitations. Firstly, the ideal study would perform imaging investigations at the time of the onset of APAC, but this was not possible due to the presence of corneal edema. If SD-OCT images were obtainable at the time of the APAC attack, the change in the ONH parameters might have been more distinct and the effect of the change in glaucoma development might have been clearer. Secondly, our sample may have been too small to detect factors that exerted small effects on glaucoma progression. Thirdly, the participants might have experienced episodes of intermittent angle closure or had a pre-existent chronic angle closure before the APAC episode. Thus, although eyes with pre-existing diagnosis of glaucoma were excluded, some individuals could have had ongoing RNFL damage before the acute attack. This may have influenced the interpretation of the results relating to RNFL changes after APAC. Since the RNFL thickness before APAC attack was not known, the progressive RNFL loss could not be determined based on the amount of RNFL loss relative to its baseline status (i.e., the rate of RNFL thinning), but was defined based on the SD-OCT findings at final examination. If the amount of RNFL loss during the follow-up had been considered for defining the progressive RNFL loss, it could not have been differentiated from the resolution of RNFL swelling after APAC attack. Fourthly, 25 of the 30 patients underwent cataract surgery during the follow-up period, which might have influenced the quality of SD-OCT images and caused measurement. However, most of the eyes had mild to moderate degree of cataract, and so any effects on the results might have been minimal. Fifthly, gonioscopy was not performed at all FU points in all patients, thus could not be considered in the analyses. Lastly, the information on the onset of symptom was obtained by self-reporting, which might not have been reliable.

In conclusion, early short-term reductions of the PLT and LCD and an overall long-term decrease in the peripapillary RNFL were observed during a 1-year follow-up after an APAC attack. A longer duration from the symptom onset to receiving LI treatment and a larger LCD reduction during the follow-up were associated with the conversion to an abnormal OCT RNFL thickness. The LCD reduction may indicate that a significant level of IOP-induced stress had been imposed on the ONH at the time of APAC episode. Glaucomatous progression should be suspected in eyes showing LCD reduction after the APAC remission.

## Supporting Information

S1 Table Data for all of the participants. (XLSX)

## References

[1] Aung T, Looi AL, Chew PT. (2001) The visual field following acute primary angle closure. Acta Ophthalmol Scand 79: 298–300. 11401643 10.1034/j.1600-0420.2001.790318.x

[2] Aung T, Friedman DS, Chew PT, Ang LP, Gazzard G, Lai YF, et al (2004) Long-term outcomes in asians after acute primary angle closure. Ophthalmology 111: 1464–1469. 10.1016/j.ophtha.2003.12.061 15288972

[3] Chen YJ, Tai MC, Cheng JH, Chen JT, Chen YH, Lu DW. (2012) The longitudinal changes of the visual field in an Asian population with primary angle-closure glaucoma with and without an acute attack. J Ocul Pharmacol Ther 28: 529–535. 10.1089/jop.2012.0006 22690869

[4] Tan AM, Loon SC, Chew PT. (2009) Outcomes following acute primary angle closure in an Asian population. Clin Experiment Ophthalmol 37: 467–472. 10.1111/j.1442-9071.2009.02060.x 19624342

[5] Shen SY, Baskaran M, Fong AC, Chan YH, Lim LS, Husain R, et al (2006) Changes in the optic disc after acute primary angle closure. Ophthalmology 113: 924–929. 10.1016/j.ophtha.2006.01.070 16751035

[6] Quek DT, Koh VT, Tan GS, Perera SA, Wong TT, Aung T. (2011) Blindness and long-term progression of visual field defects in chinese patients with primary angle-closure glaucoma. Am J Ophthalmol 152: 463–469. 10.1016/j.ajo.2011.02.023 21676375

[7] Thomas R, Parikh R, Muliyil J, Kumar RS. (2003) Five-year risk of progression of primary angle closure to primary angle closure glaucoma: a population-based study. Acta Ophthalmol Scand 81: 480–485. 14510795 10.1034/j.1600-0420.2003.00135.x

[8] Aung T, Husain R, Gazzard G, Chan YH, Devereux JG, Hoh ST, et al (2004) Changes in retinal nerve fiber layer thickness after acute primary angle closure. Ophthalmology 111: 1475–1479. 10.1016/j.ophtha.2003.12.055 15288974

[9] Tsai JC. (2006) Optical coherence tomography measurement of retinal nerve fiber layer after acute primary angle closure with normal visual field. Am J Ophthalmol 141: 970–972. 10.1016/j.ajo.2005.12.020 16678526

[10] Chew SS, Vasudevan S, Patel HY, Gurria LU, Kerr NM, Gamble G, et al (2011) Acute primary angle closure attack does not cause an increased cup-to-disc ratio. Ophthalmology 118: 254–259. 10.1016/j.ophtha.2010.06.026 20884056

[11] Burgoyne CF, Downs JC. (2008) Premise and prediction-how optic nerve head biomechanics underlies the susceptibility and clinical behavior of the aged optic nerve head. J Glaucoma 17: 318–328. 10.1097/IJG.0b013e31815a343b 18552618 PMC2777521

[12] Burgoyne CF, Downs JC, Bellezza AJ, Suh JK, Hart RT. (2005) The optic nerve head as a biomechanical structure: a new paradigm for understanding the role of IOP-related stress and strain in the pathophysiology of glaucomatous optic nerve head damage. Prog Retin Eye Res 24: 39–73. 10.1016/j.preteyeres.2004.06.001 15555526

[13] Burgoyne CF. (2011) A biomechanical paradigm for axonal insult within the optic nerve head in aging and glaucoma. Exp Eye Res 93: 120–132. 10.1016/j.exer.2010.09.005 20849846 PMC3128181

[14] Park SC, Brumm J, Furlanetto RL, Netto C, Liu Y, Tello C, et al (2015) Lamina cribrosa depth in different stages of glaucoma. Invest Ophthalmol Vis Sci 56: 2059–2064. 10.1167/iovs.14-15540 25722212

[15] Ren R, Yang H, Gardiner SK, Fortune B, Hardin C, Demirel S, et al (2014) Anterior lamina cribrosa surface depth, age, and visual field sensitivity in the Portland Progression Project. Invest Ophthalmol Vis Sci 55: 1531–1539. 10.1167/iovs.13-13382 24474264 PMC3954157

[16] Inoue R, Hangai M, Kotera Y, Nakanishi H, Mori S, Morishita S, et al (2009) Three-dimensional high-speed optical coherence tomography imaging of lamina cribrosa in glaucoma. Ophthalmology 116: 214–222. 10.1016/j.ophtha.2008.09.008 19091413

[17] Park HY, Jeon SH, Park CK. (2012) Enhanced depth imaging detects lamina cribrosa thickness differences in normal tension glaucoma and primary open-angle glaucoma. Ophthalmology 119: 10–20. 10.1016/j.ophtha.2011.07.033 22015382

[18] You JY, Park SC, Su D, Teng CC, Liebmann JM, Ritch R. (2013) Focal lamina cribrosa defects associated with glaucomatous rim thinning and acquired pits. JAMA Ophthalmol 131: 314–320. 10.1001/jamaophthalmol.2013.1926 23370812

[19] Faridi OS, Park SC, Kabadi R, Su D, De Moraes CG, Liebmann JM, et al (2014) Effect of focal lamina cribrosa defect on glaucomatous visual field progression. Ophthalmology 121: 1524–1530. 10.1016/j.ophtha.2014.02.017 24697910

[20] Lee SH, Lee EJ, Kim TW. (2015) Structural Characteristics of the Acquired Optic Disc Pit and the Rate of Progressive Retinal Nerve Fiber Layer Thinning in Primary Open-Angle Glaucoma. JAMA Ophthalmol. 10.1001/jamaophthalmol.2015.2453 26247160

[21] Sullivan-Mee M, Patel NB, Pensyl D, Qualls C. (2015) Relationship Between Juxtapapillary Choroidal Volume and Beta-Zone Parapapillary Atrophy in Eyes With and Without Primary Open-Angle Glaucoma. Am J Ophthalmol 160: 637–647 e631. 10.1016/j.ajo.2015.06.024 26144700 PMC4569512

[22] Lee EJ, Kim TW. (2015) Lamina Cribrosa Reversal after Trabeculectomy and the Rate of Progressive Retinal Nerve Fiber Layer Thinning. Ophthalmology. 10.1016/j.ophtha.2015.07.020 26298719

[23] Lee EJ, Kim TW, Kim M, Kim H. (2015) Influence of lamina cribrosa thickness and depth on the rate of progressive retinal nerve fiber layer thinning. Ophthalmology 122: 721–729. 10.1016/j.ophtha.2014.10.007 25433610

[24] Park HY, Shin HY, Jung KI, Park CK. (2014) Changes in the lamina and prelamina after intraocular pressure reduction in patients with primary open-angle glaucoma and acute primary angle-closure. Invest Ophthalmol Vis Sci 55: 233–239. 10.1167/iovs.12-10329 24204049

[25] Jiang R, Xu L, Liu X, Chen JD, Jonas JB, Wang YX. (2015) Optic nerve head changes after short-term intraocular pressure elevation in acute primary angle-closure suspects. Ophthalmology 122: 730–737. 10.1016/j.ophtha.2014.11.008 25556115

[26] Van Herick W, Shaffer RN, Schwartz A. (1969) Estimation of width of angle of anterior chamber. Incidence and significance of the narrow angle. Am J Ophthalmol 68: 626–629. 5344324 10.1016/0002-9394(69)91241-0

[27] Spaide RF, Koizumi H, Pozzoni MC. (2008) Enhanced depth imaging spectral-domain optical coherence tomography. American journal of ophthalmology 146: 496–500. Epub 2008/07/22. 10.1016/j.ajo.2008.05.032 18639219

[28] Lee EJ, Kim TW, Weinreb RN, Park KH, Kim SH, Kim DM. (2011) Visualization of the lamina cribrosa using enhanced depth imaging spectral-domain optical coherence tomography. American journal of ophthalmology 152: 87–95 e81. Epub 2011/05/17. 10.1016/j.ajo.2011.01.024 21570046

[29] Wu H, de Boer JF, Chen TC. (2011) Reproducibility of retinal nerve fiber layer thickness measurements using spectral domain optical coherence tomography. Journal of glaucoma 20: 470–476. Epub 2010/09/21. 10.1097/IJG.0b013e3181f3eb64 20852437 PMC3500562

[30] Alasil T, Wang K, Keane PA, Lee H, Baniasadi N, de Boer JF, et al (2013) Analysis of normal retinal nerve fiber layer thickness by age, sex, and race using spectral domain optical coherence tomography. Journal of glaucoma 22: 532–541. Epub 2012/05/03. 10.1097/IJG.0b013e318255bb4a 22549477

[31] Lee EJ, Kim TW, Weinreb RN, Kim H. (2013) Reversal of lamina cribrosa displacement after intraocular pressure reduction in open-angle glaucoma. Ophthalmology 120: 553–559. 10.1016/j.ophtha.2012.08.047 23218823

[32] Lee EJ, Kim TW, Weinreb RN. (2013) Variation of lamina cribrosa depth following trabeculectomy. Investigative ophthalmology & visual science 54: 5392–5399. 23838772 10.1167/iovs.13-12205

[33] Lee EJ, Kim TW, Weinreb RN. (2012) Reversal of lamina cribrosa displacement and thickness after trabeculectomy in glaucoma. Ophthalmology 119: 1359–1366. 10.1016/j.ophtha.2012.01.034 22464141

[34] Johnstone J, Fazio M, Rojananuangnit K, Smith B, Clark M, Downs C, et al (2014) Variation of the axial location of Bruch's membrane opening with age, choroidal thickness, and race. Invest Ophthalmol Vis Sci 55: 2004–2009. 10.1167/iovs.13-12937 24595390 PMC3973189

[35] Lee KM, Kim TW, Weinreb RN, Lee EJ, Girard MJ, Mari JM. (2014) Anterior lamina cribrosa insertion in primary open-angle glaucoma patients and healthy subjects. PLoS One 9: e114935 10.1371/journal.pone.0114935 25531761 PMC4273977

[36] Man X, Chan NC, Baig N, Kwong YY, Leung DY, Li FC, et al (2015) Anatomical effects of clear lens extraction by phacoemulsification versus trabeculectomy on anterior chamber drainage angle in primary angle-closure glaucoma (PACG) patients. Graefes Arch Clin Exp Ophthalmol 253: 773–778. 10.1007/s00417-015-2936-z 25644619

[37] Tojo N, Otsuka M, Miyakoshi A, Fujita K, Hayashi A. (2014) Improvement of fluctuations of intraocular pressure after cataract surgery in primary angle closure glaucoma patients. Graefes Arch Clin Exp Ophthalmol 252: 1463–1468. 10.1007/s00417-014-2666-7 24862301

[38] Dada T, Rathi A, Angmo D, Agarwal T, Vanathi M, Khokhar SK, et al (2015) Clinical outcomes of clear lens extraction in eyes with primary angle closure. J Cataract Refract Surg 41: 1470–1477. 10.1016/j.jcrs.2014.10.029 26287886

[39] Tsai JC, Lin PW, Teng MC, Lai IC. (2007) Longitudinal changes in retinal nerve fiber layer thickness after acute primary angle closure measured with optical coherence tomography. Invest Ophthalmol Vis Sci 48: 1659–1664. 10.1167/iovs.06-0950 17389497

[40] Fang AW, Qu J, Li LP, Ji BL. (2007) Measurement of retinal nerve fiber layer in primary acute angle closure glaucoma by optical coherence tomography. J Glaucoma 16: 178–184. 10.1097/IJG.0b013e31802d6dd8 17473726

[41] Liu X, Li M, Zhong YM, Xiao H, Huang JJ, Kong XY. (2010) Damage patterns of retinal nerve fiber layer in acute and chronic intraocular pressure elevation in primary angle closure glaucoma. Int J Ophthalmol 3: 152–157. 10.3980/j.issn.2222-3959.2010.02.14 22553541 PMC3340777

[42] Sng CC, See JS, Ngo CS, Singh M, Chan YH, Aquino MC, et al (2011) Changes in retinal nerve fibre layer, optic nerve head morphology, and visual field after acute primary angle closure. Eye (Lond) 25: 619–625. 21436844 10.1038/eye.2011.31 PMC3171266

[43] Jonas JB, Hayreh SS, Tao Y, Papastathopoulos KI, Rensch F. (2012) Optic nerve head change in non-arteritic anterior ischemic optic neuropathy and its influence on visual outcome. PLoS One 7: e37499 10.1371/journal.pone.0037499 22629408 PMC3357379

[44] Kaur C, Sivakumar V, Foulds WS. (2006) Early response of neurons and glial cells to hypoxia in the retina. Invest Ophthalmol Vis Sci 47: 1126–1141. 10.1167/iovs.05-0518 16505051

[45] Bellezza AJ, Rintalan CJ, Thompson HW, Downs JC, Hart RT, Burgoyne CF. (2003) Anterior scleral canal geometry in pressurised (IOP 10) and non-pressurised (IOP 0) normal monkey eyes. Br J Ophthalmol 87: 1284–1290. 14507767 10.1136/bjo.87.10.1284 PMC1920775

[46] Hayreh SS. (1969) Blood supply of the optic nerve head and its role in optic atrophy, glaucoma, and oedema of the optic disc. Br J Ophthalmol 53: 721–748. 4982590 10.1136/bjo.53.11.721 PMC506749

[47] Quigley HA, Anderson DR. (1977) Distribution of axonal transport blockade by acute intraocular pressure elevation in the primate optic nerve head. Invest Ophthalmol Vis Sci 16: 640–644. 68942

[48] Quigley HA, Guy J, Anderson DR. (1979) Blockade of rapid axonal transport. Effect of intraocular pressure elevation in primate optic nerve. Arch Ophthalmol 97: 525–531. 84662 10.1001/archopht.1979.01020010269018

